# Exploring the Reaction Mechanism of Polyethylene Terephthalate Biodegradation through QM/MM Approach

**DOI:** 10.1021/acs.jpcb.4c02207

**Published:** 2024-07-29

**Authors:** Alberto M. dos Santos, Clauber H. S. da Costa, Pedro H. A. Silva, Munir S. Skaf, Jerônimo Lameira

**Affiliations:** † Institute of Chemistry and Centre for Computer in Engineering and Sciences, University of Campinas (UNICAMP), Campinas 13084-862, Sao Paulo, Brazil; ‡ Institute of Biological Sciences, Federal University of Para, 66075-110 Belem, Para, Brazil

## Abstract

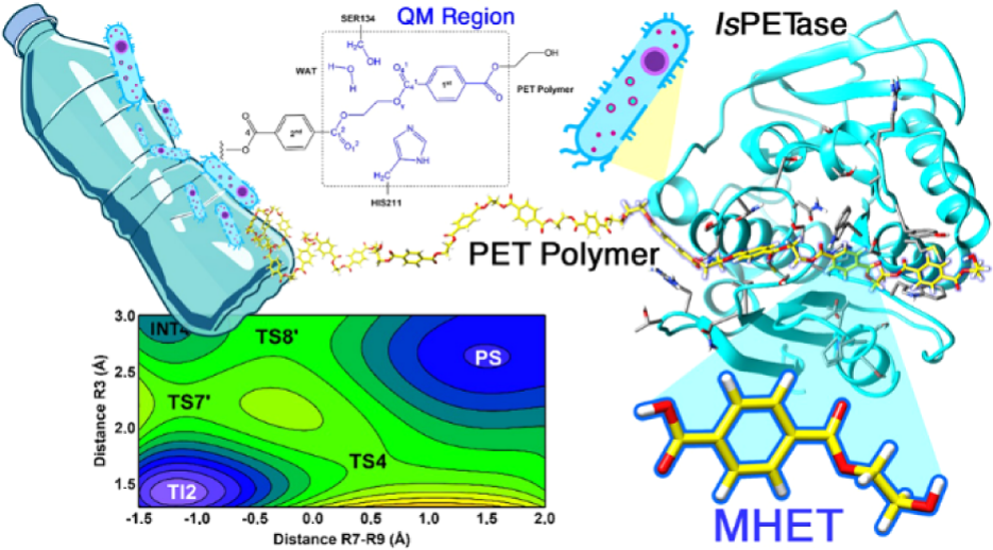

The enzyme PETase from*Ideonella sakaiensis* (*Is*PETase) strain 201-F6 can catalyze the hydrolysis of polyethylene terephthalate (PET), mainly converting it into mono(2-hydroxyethyl) terephthalic acid (MHET). In this study, we used quantum mechanics/molecular mechanics (QM/MM) simulations to explore the molecular details of the catalytic reaction mechanism of *Is*PETase in the formation of MHET. The QM region was described with AM1d/PhoT and M06-2*X*/6-31+G(d,p) potential. QM/MM simulations unveil the complete enzymatic PET hydrolysis mechanism and identify two possible reaction pathways for acylation and deacylation steps. The barrier obtained at M06-2*X*/6-31+G(d,p)/MM potential for the deacylation step corresponds to 20.4 kcal/mol, aligning with the experimental value of 18 kcal/mol. Our findings indicate that deacylation is the rate-limiting step of the process. Furthermore, per-residue interaction energy contributions revealed unfavorable contributions to the transition state of amino acids located at positions 200–230, suggesting potential sites for targeted mutations. These results can contribute to the development of more active and selective enzymes for PET depolymerization.

## Introduction

The advent of polyethylene terephthalate (PET) has significantly transformed modern human civilization, offering versatile applications across the plastics industry. However, synthetic polymers like PET-based plastic waste continue to present environmental challenges, threatening ecosystems and biodiversity due to their strong resistance to biodegradation.^1−5^

Enzymatic hydrolysis offers an alternative way to selectively generate monomers under mild conditions, thereby circumventing the use of chemical waste such as organic solvents.^6^*Ideonella sakaiensis* strain 201-F6 was recently discovered with the ability to degrade and use synthetic polymers, such as PET, as its major energy and carbon source.^7^ This discovery has paved the way for novel scientific investigations aimed at identifying eco-friendly alternatives for managing plastic waste through enzymatic recycling at moderate temperatures (20 to 45 °C).^8−11^

The Yoshida group demonstrated that *I. sakaiensis* expresses two closely related enzymes involved with PET degradation.^7,12^ The first enzyme is named PETase (PET-digesting enzyme), which converts PET to mono(2-hydroxyethyl) terephthalic acid (MHET), bis(2-hydroxyethyl)-TPA (BHET), and terephthalic acid (TPA) as products. The second enzyme is the MHETase (MHET-digesting enzyme), which further converts MHET into two monomers: ethylene glycol (EG) and TPA.^13^ Structural and evolutionary studies of *I. sakaiensis* PETase (*Is*PETase) have shown that its structure resembles α/β-hydrolase enzymes.^14−16^ The α/β-hydrolase family includes lipases and cutinases, which catalyze the hydrolysis of fatty acids and cutin, respectively.^14−16^

Several studies have proposed a molecular mechanism for PET degradation catalyzed by *Is*PETase.^14,17,18^ Moliner and colleagues investigated the atomic-level mechanism of the *Is*PETase enzyme using computational methods, outlining a four-step process for *Is*PETase that involved two or three monomers and a semiempirical potential with DFT corrections. They have found that acylation and deacylation take place in a stepwise mechanism.^19^ Subsequently, Carola and co-workers conducted a similar study on *Is*PETase, suggesting that both acylation and deacylation proceed through a single, associative, tetrahedral transition state in a concerted yet asynchronous manner.^20^ Both contributions^19,20^ have proposed that acylation is the rate-limiting step of the process. On the other hand, Knott et al. demonstrated that the deacylation step is the rate-limiting catalytic mechanism of MHETase.^21^ They have also proposed that MHETase’s catalytic mechanism occurs without stable tetrahedral intermediates, with acyl-enzyme intermediate formation (acylation) and hydrolysis (deacylation) taking place as individual steps.^21^ These studies employed benchmark simulations using free energy surfaces and a quantum mechanics/molecular mechanics (QM/MM) approach to explore PET degradation catalyzed by *Is*PETase and MHETase.^19−21^

Previously, we investigated the conformational dynamics of *Is*PETase in response to PET binding, highlighting details of its structural adaptability and regions of flexibility that could be targeted to enhance enzyme stability and efficacy.^22^ Here, we present a QM/MM study of PET degradation by *Is*PETase, focusing on the formation of the product MHET using a PET tetramer. We have compared our results with recently published computational studies^19−21^ and further developed the current general model of the catalytic mechanism of *Is*PETase.

## Theoretical Methods

**Scheme 1:**
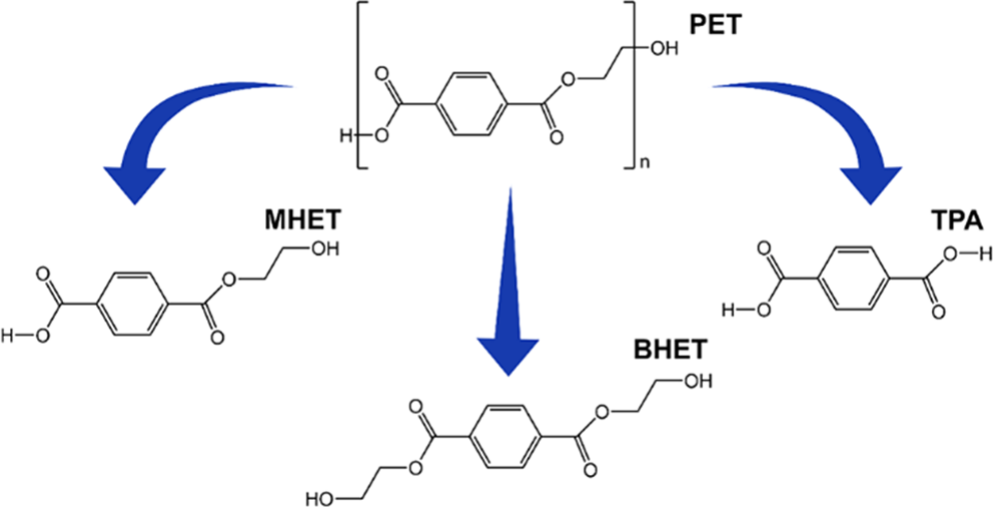
*Is*PETase Catalyzes the Hydrolysis of PET into MHET and BHET

As described in the Introduction section, *Is*PETase catalyzes PET degradation into MHET, BHET, and TPA (Scheme 1), with MHET as the major product of *Is*PETase.^7^ The PET-binding mode has been predicted mostly through computational methods.^16,23,24^ Recently, we proposed the binding mode of PET into the active site of *Is*PETase and provided molecular details of PET’s main interaction using molecular docking and molecular dynamics (MD) simulations.^22^ Here, we used the PET-*Is*PETase complex described before^22^ as a starting point to explore the catalytic mechanism of *Is*PETase for PET degradation into MHET and BHET. The system consists of the tetramer (2-hydroxyethyl-(monohydroxyethyl terephthalate)_4_, 2-HE(MHET)_2_) in the complex with *Is*PETase, additional details regarding the initial structure used for the simulation can be found elsewhere.^22^

### The Free Energy Surface (FES)

**Scheme 2:**
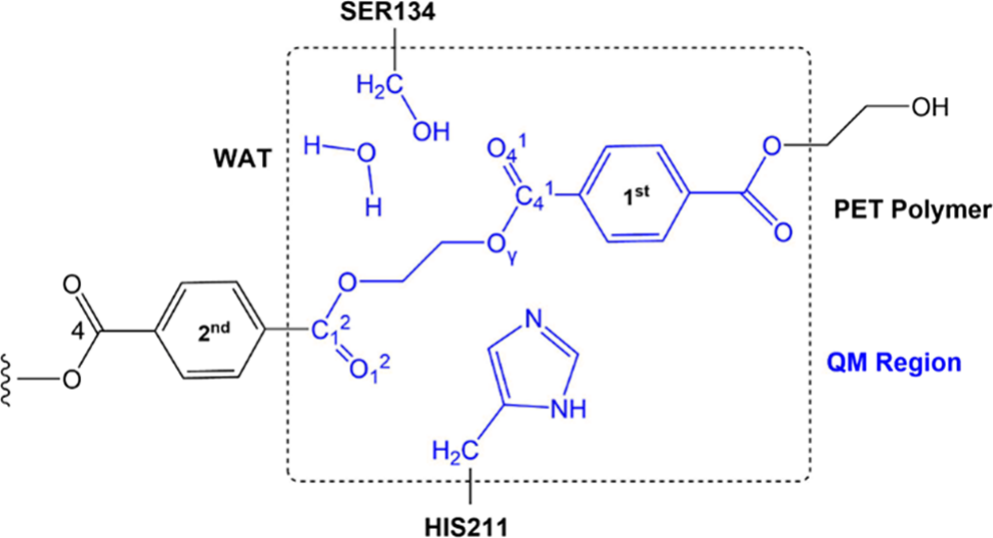
Quantum Region Used in QM/MM Simulations

To obtain the FES associated with MHET formation, we have used the weighted histogram analysis method (WHAM) combined with the umbrella sampling approach^25^ as implemented in the pDynamo program.^26^ The PMF calculation requires a series of molecular dynamics simulations in which the distinguished reaction coordinate variable, ξ, is constrained around particular values.^27^ In the QM/MM approach, a small part of the system (ligand/substrate species) is described by quantum mechanics, while MM force fields represent the protein and solvent environment.^28^ The 2D PMFs were obtained for the mechanism of MHET formation. A wide range of semiempirical methods are used within hybrid QM/MM simulations to study different systems, including metallic nanoparticles^29^ and enzymatic reactions.^30^ Semiempirical potentials can be based on s and p orbitals (MNDO, AM1, PM3, and RM1) or also included orbitals, either based on approximations to Hartree–Fock theory (MNDO/d, PM6, and AM1/d-PhoT) or derived from density functional theory (DFTB3).^30^ In this study, the AM1d/PhoT potential was used to describe the QM region. The atomic coordinates of the atoms involved in the reaction were restrained by a harmonic umbrella potential of 50 kcal·mol^–1^·Å^2^. For the hybrid QM/MM calculations, the atoms of the PET, a water molecule, and the side chains of Ser134 and His211 residues were selected to be treated by QM using a semiempirical AM1/d-PhoT^31^ Hamiltonian. The other atoms of the system, protein, and water molecules were described using the CHARMM/TIP3P^35^ force fields, respectively. The number of QM atoms then resulted in 44, while the final system contains 32,428 atoms, respectively. It is important to note that the QM region (Scheme 2) used in this work includes a smaller number of residues than in previous computational studies.^19,20^ The simulation with a small QM region is computationally less demanding, and the size of the QM region may have little influence on the single-point QM/MM calculations for studies involving enzymatic catalysis.^36,37^

Initially, QM/MM Langevin-Verlet MD at 300 K and in a canonical thermodynamic ensemble (NVT) were used to equilibrate the system. Due to the number of degrees of freedom, any residue 20 Å apart from any of the reactants’ atoms was selected to be frozen in the remaining calculations. Cut-offs for the nonbonding interactions were applied using a switching scheme within a range radius of 14.5 to 16.0 Å. Afterward, the system was equilibrated by means of 2.0 ns of QM/MM MD at a temperature of 300 K. The computed RMSD for the protein during the last 1 ns renders a value always below 0.9 Å. Furthermore, the temperature RMS along the different equilibration steps was always lower than 2.5 K, and the potential energy variation coefficient during the dynamics simulations was never higher than 0.3%.

**Scheme 3:**
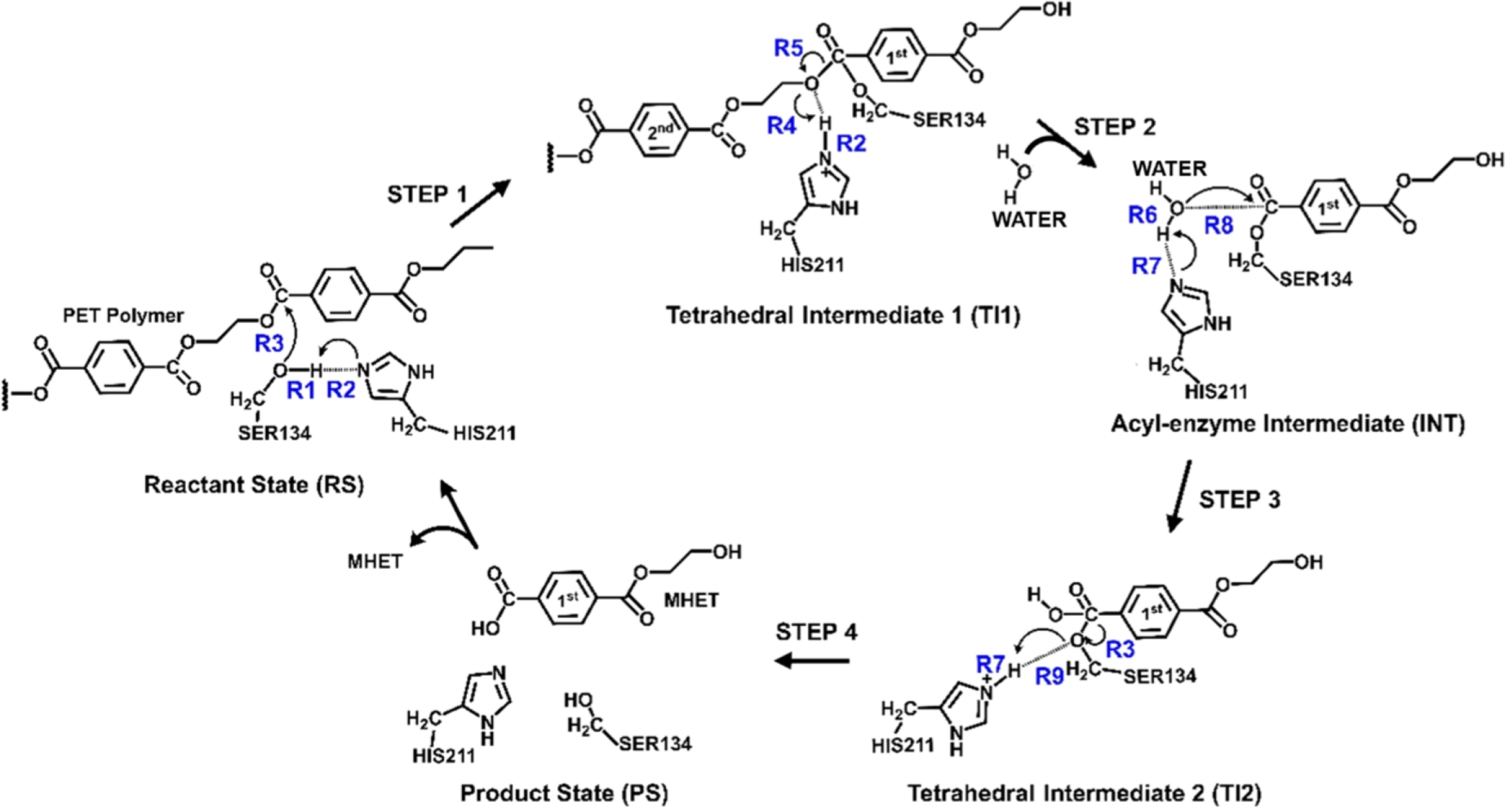
Reaction Coordinates Used to Investigate the PET Degradation Reaction Mechanism

A total of 40 simulations were performed at different values of the R1-R2 antisymmetric combination of distances (ranging from −2.0 to 2.0 Å, see Scheme 3), with an umbrella force constant of 50 kcal.mol^–1^·Å^–2^ applied to this distinguished reaction coordinate. In addition, 40 simulations were performed at different values of R3 (ranging from 1.0 to 3.0 Å, see Scheme 3), also with an umbrella force constant of 50 kcal.mol^–1^·Å^–2^ on this combination of distances. Consequently, 1600 simulation windows were needed to obtain the 2D-PMF for Step 1 of the PET degradation mechanism of *Is*PETase (Scheme 3). R1-R2 described the proton transfer from Ser134 to His211, and R3 described the nucleophilic attack of Ser134 on the C_4_^1^ of PET. The same protocol was used for Step 2, where the antisymmetric combination of distances R2-R4 and the distance R5 was used to describe, respectively, the proton transfer from His211 to the oxygen O_*y*_ of PET and the PET C_4_^1^-O_*y*_ bond breakdown. For Step 3, the antisymmetric combination of distances R6-R7 and the distance R8 was used to describe, respectively, the proton transfer from water to histidine and the nucleophilic attack of water on the C_4_^1^ of PET. For Step 4, the antisymmetric combination of distances R7-R9 and the distance R3 were used to describe, respectively, the proton transfer from His211 to Ser134 and the C_4_^1^-O_Ser134_ bond breakdown. The values of the variables sampled during the simulations were then pieced together to construct a full distribution function, from which the 2D-PMF was obtained. In each window, 20 ps of relaxation were followed by 20 ps of production with a time step of 0.5 fs due to the nature of the chemical step involving a hydrogen transfer. The Verlet algorithm was used to update the velocities. It is important to note that the coordinates considered in the PMF are described in Scheme 3. The transition states were identified as saddle points (see Supporting Information (SI)).

Since the use of QM/MM calculations in the evaluation of free energy is commonly restricted to semiempirical Hamiltonians due to the large number of gradient vector evaluations, we initiated our analysis with a semiempirical potential. Then, the error associated with this quantum level of theory was reduced by including correction terms. The corrections were applied by subtracting the energy calculated for the QM region using a semiempirical level and adding the energy from the DFT potential.^38^ The QM region was described with the M06-2X^38^ functional with the 6-31+G(d,p) basis set. The TS-like conformations obtained from AM1/d-PhoT/MM MD simulations were used as the starting geometries for the correction energy at the DFT level. The QM corrections were performed using the ORCA 5.0.3 quantum chemistry program suite^39^ and the pDynamo program.^26^ The same high-level QM corrections have been applied previously.^40^

### Per-residue Interaction Energy Calculations

Furthermore, we investigated how active site residues stabilize or destabilize the species present along the free energy profile, analyzing the interaction energy of each protein residue with the ligand (TS and RS). There are different ways of exploring interaction energy per residue using the QM/MM approach. For instance, Quantum Mechanics-Poisson–Boltzmann Surface Area (QM-PBSA).^41^ While insightful, the computational demands posed significant challenges for larger sampling endeavors and for exploring the energy interaction between the protein and different species along the reaction path. Other strategies employed QM/MM calculations to elucidate the interactions between proteins and ligands through the Interacting Quantum Atoms approach.^42^ Despite offering novel insights into utilizing QM and QM/MM calculations for analyzing energy contributions to protein structure and binding, these methods often overlook transition state structures and the roles of specific residues in each step of chemical reactions. Here, we have made use of a hybrid M06-2X/CHARMM36m method based on a semiclassical approach to obtain the interaction energy between the substrates and transition states, obtained from the mechanism and the environment. This interaction energy is evaluated as the difference between the QM/MM energy and the energies of the separated, noninteracting QM and MM subsystems with the same geometry. Considering that the MM part is described using a nonpolarizable potential, the interaction energy contribution of each residue of the protein is given by the following expression:
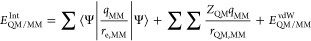
1

This interaction energy can be exactly decomposed into a sum over residues, provided that the polarized wave function (Ψ) is employed to evaluate this energy contribution. It is important to point out that although the structures used to calculate the per-residue energy contribution were obtained from the reaction coordinates describing the mechanism, the QM region used in the decomposition analysis was the whole PET molecule (98 atoms) for TS1 and RS and the PET molecule and water (101 atoms), without His211 and Ser134 in the QM region. The same strategy has been used in previous contributions.^43,44^

## Results and Discussion

### Free Energy Surface for Exploring Reaction Paths for PET Hydrolysis

*Is*PETase, which belongs to the serine hydrolase family, shares a similar molecular mechanism with cutinases,^45^ involving the formation of an acyl-enzyme intermediate in the first step and a nucleophilic attack by a water molecule in the second step.^16^ The PET hydrolysis mechanism was investigated using ONIOM calculations in previous computational studies.^45,46^ Despite the limitations of ONIOM calculations, such as the inability to account for the dynamic behavior of the enzyme, it is possible to reasonably estimate the activation barrier associated with each step of the molecular mechanism. The potential energy surfaces obtained using QM/MM potentials can also be used to explore enzymatic mechanisms, albeit with the same limitations as ONIOM calculations.^48^ Note that Determining the activation energy of the reaction catalyzed by *Is*PETase would be extremely resource-intensive when using free energy surfaces acquired through umbrella sampling at the DFT/MM potential. Therefore, we used QM/MM and molecular dynamics simulations with the semiempirical potential AM1/d-PhoT^31^ to describe the QM region. This semiempirical potential is parametrized to reproduce high-level density-functional results and have been used for study different enzymatic reaction mechanisms.^32−34^

Understanding the catalytic mechanism of *Is*PETase and investigating the impact of key mutations in this enzyme are crucial steps for enhancing enzymatic catalysis. It is worth noting that examining the effects of proposed mutations on the activation energy of the reaction catalyzed by *Is*PETase would be extremely resource-intensive when using free energy surfaces acquired through umbrella sampling at the DFT/MM potential, even for a small QM region. In simpler terms, the computational expenses tied to assessing free energy surfaces in proteins make it challenging to derive dependable surfaces for enzymatic reactions with both a sufficiently high *ab initio* level and adequate sampling. One alternative for analyzing the influence of mutations on the activation energy of the reaction catalyzed by *Is*PETase could involve the application of Empirical Valence Bond (EVB) methods,^49^ which is a simple and useful tool for obtaining activation energy in a protein environment. On the other hand, this approach requires a well-calibrated EVB surface in solution.

**Figure 1:**
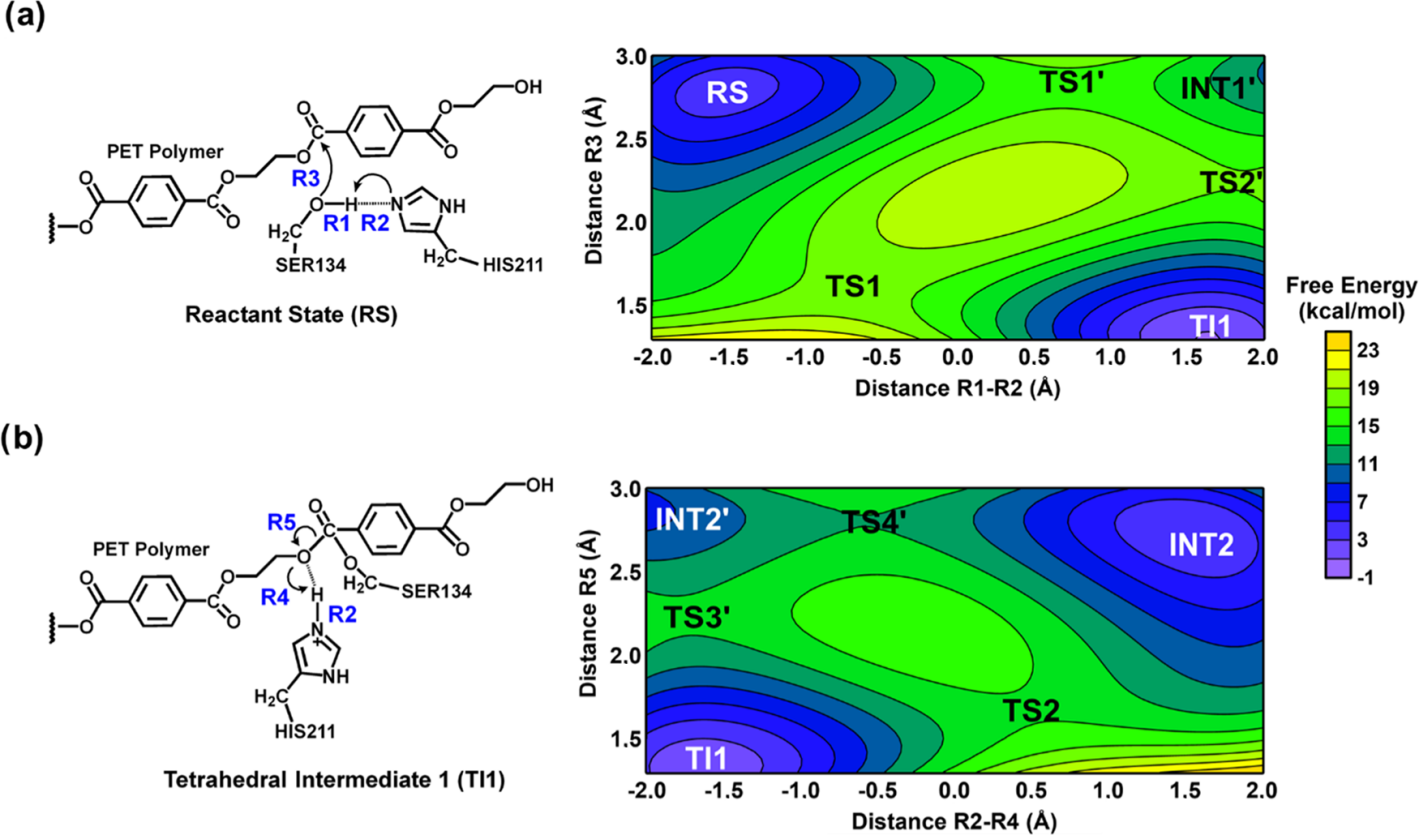
Free energy surface (FES) at AM1/d-PhoT/MM potential for mechanism of reaction involving *Is*PETase. The acylation is described by two steps: (a) Step 1, where the formation of tetrahedral intermediate (TI1) is observed, and (b) Step 2, where the formation of MHET (INT2) is observed. Contour interval of 2 kcal/mol.

Our goals were to compute the activation energy for the reaction catalyzed by *Is*PETase and compare it with experimental data and previous QM/MM simulation results.^14,17−21^ The combination of experimental^7,9^ and computational data offers a comprehensive understanding of the enzyme’s functions, and the identification of specific amino acid residues that contribute to the stabilization or destabilization of the transition states can reveal potential targeted mutations for enhanced enzyme activity. Here, we initiated our analysis with the Potential of Mean Force (PMF) using QM/MM potential for exploring the acylation step (Figure 1). The FES results provide a more detailed picture of the reaction mechanism, including a dynamic description of the enzymatic environment. To generate the FES, we applied the AM1/d-PhoT^31^ Hamiltonian to describe the QM region (Scheme 2) during QM/MM MD simulations, which lacks accuracy compared to *ab initio* and DFT calculations, but it is fast enough to simulate QM/MM molecular dynamics simulation and obtain a proper sampling.

Figure 1 shows the FES used to describe the first step of PET degradation. This FES describes the acylation step, where R1, R2, and R3 were used as distinguished reaction coordinates for describing the chemical reaction. R1-R2 correspond to the proton transfer from Ser134-OG to His211-NE1. Note that OG is the hydroxyl group of Ser134, and NE1 is the N of imidazole in the His211 residue. R3 corresponds to the attack of Ser134-OG on the ester carbon atom of the first subunit of the tetramer, which represents the PET polymer in our simulations. Here, this carbon atom was labeled as C_4_^1^, as proposed by reference.^20^

**Figure 2:**
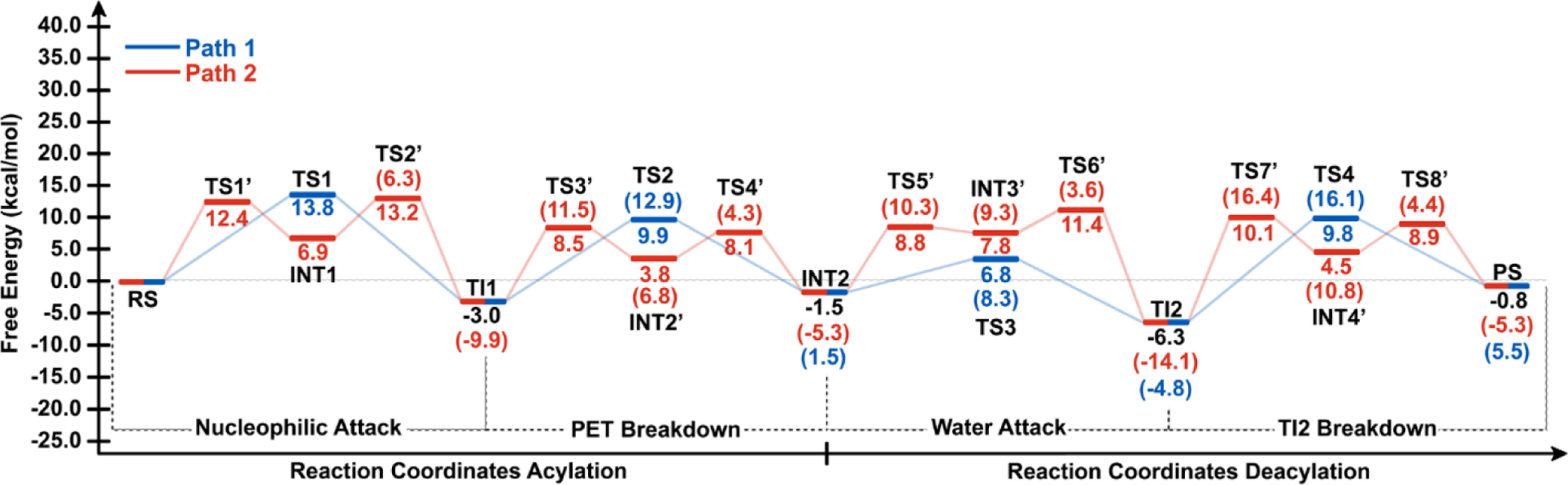
Free energies calculated at the AM1/d-PhoT/MM level for the MHET Formation. The values in parentheses represent the energy difference between the TS and its preceding intermediate, while the values outside parentheses are calculated with respect to the RS. Path 1 is in blue, and Path 2 is in red. Note that for some intermediates, two values are described for the stepwise (red) and the concerted (blue) paths. The units are in kcal/mol.

**Figure 3:**
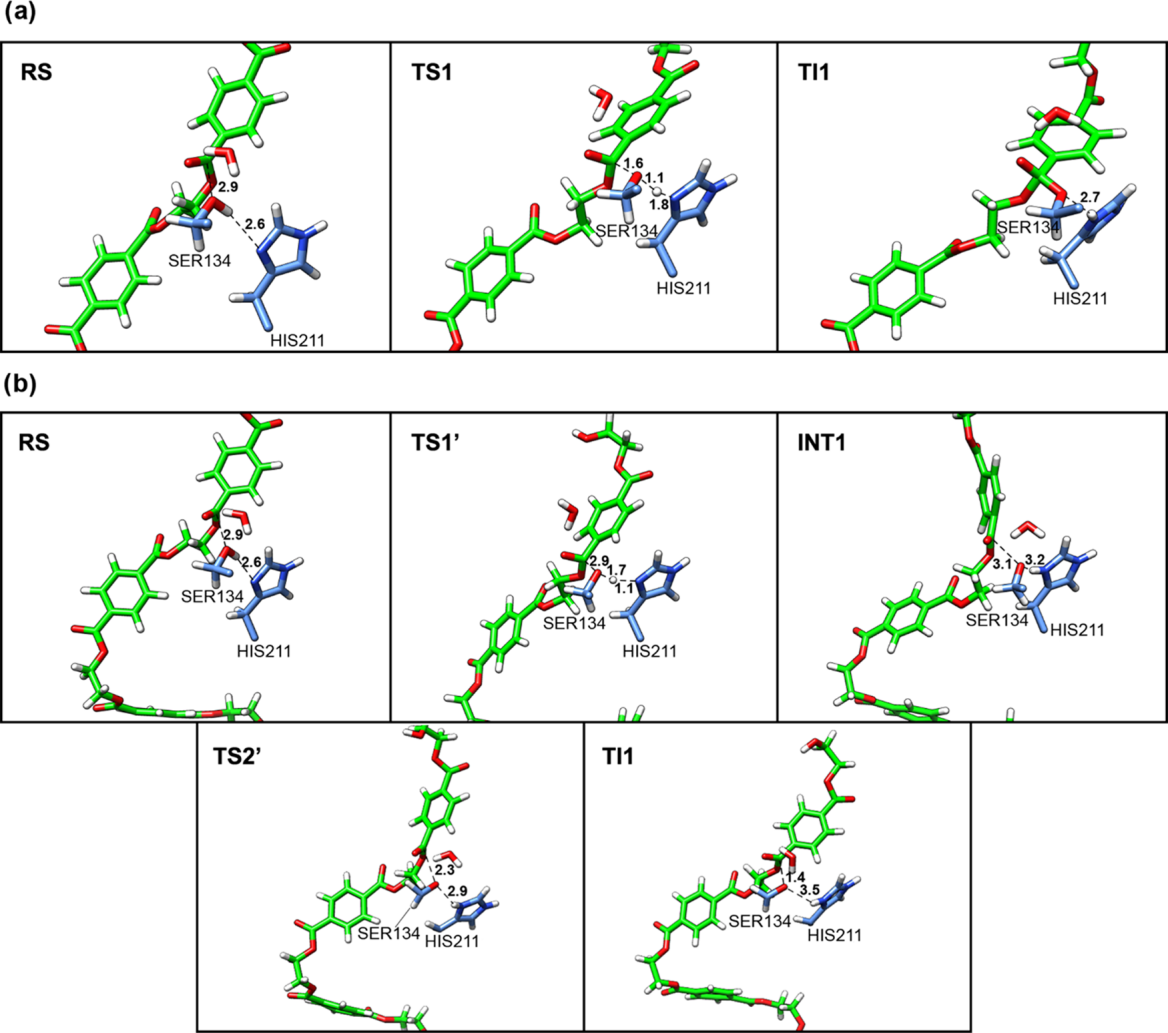
Representative structures along the 2D-PMF obtained from the QM/MM free-energy simulations for Step 2 acylation PET breakdown: (a) Path 1, concerted proposal; (b) Path 2, stepwise proposal for TI formation. Structures are labeled according to their locations along the 2D PMF.

The resulting FES obtained with AM1/d-PhoT/MM renders two possible paths for the formation of the tetrahedral intermediate (TI). Path 1 occurs through TS1 formation (Figure 1a). The free energy of activation associated with this path corresponds to 13.8 kcal/mol (see Figure 2). In TS1, the average distance between Ser134-OG and the carbon atom (C_4_^1^) corresponds to 1.6 Å, aligning with the value of 1.5 Å obtained from FES using DFT/MM as potential.^20^ The average distance between the proton of Ser134-OG and His211-NE1 is 1.8 Å, with the proton located closer to Ser134 (1.1 Å) (Figure 3a and Table S1), which indicates that this proton transfer is in an earlier stage of the process.

Path 2 (Figure 1a) occurs in a stepwise mechanism through TS1′ and TS2′ formation. The free energy of activation associated with this path corresponds to 12.4 and 6.3 kcal/mol for the first and second steps, respectively (see Figure 2). In the TS1′, the average distance between Ser134-OG and the carbon atom (C_4_^1^) corresponds to 2.98 Å, and the average distance between the proton of Ser134-OG and His211-NE1 is 1.1 Å, with the proton located closer to His211 (Figure 3b and Table S2). This result indicates a very advanced stage for proton transfer and suggests that the nucleophilic attack of Ser134 on the carbon atom (C_4_^1^) may occur after the deprotonation of Ser134 by His211. Path 2 agrees with *Is*PETase’s recently proposed mechanism for the acylation step of PET degradation.^20^ In the TS2′, the average distance between Ser134-OG and the carbon atom (C_4_^1^) corresponds to 2.3 Å (Figure 3b and Table S2). The product of this step is the tetrahedral intermediate (TI1 on Figure 1). It is worth noting that previous work has also suggested that the formation of tetrahedral intermediate in the acylation stage may occur through a stepwise path.^46^

**Figure 4:**
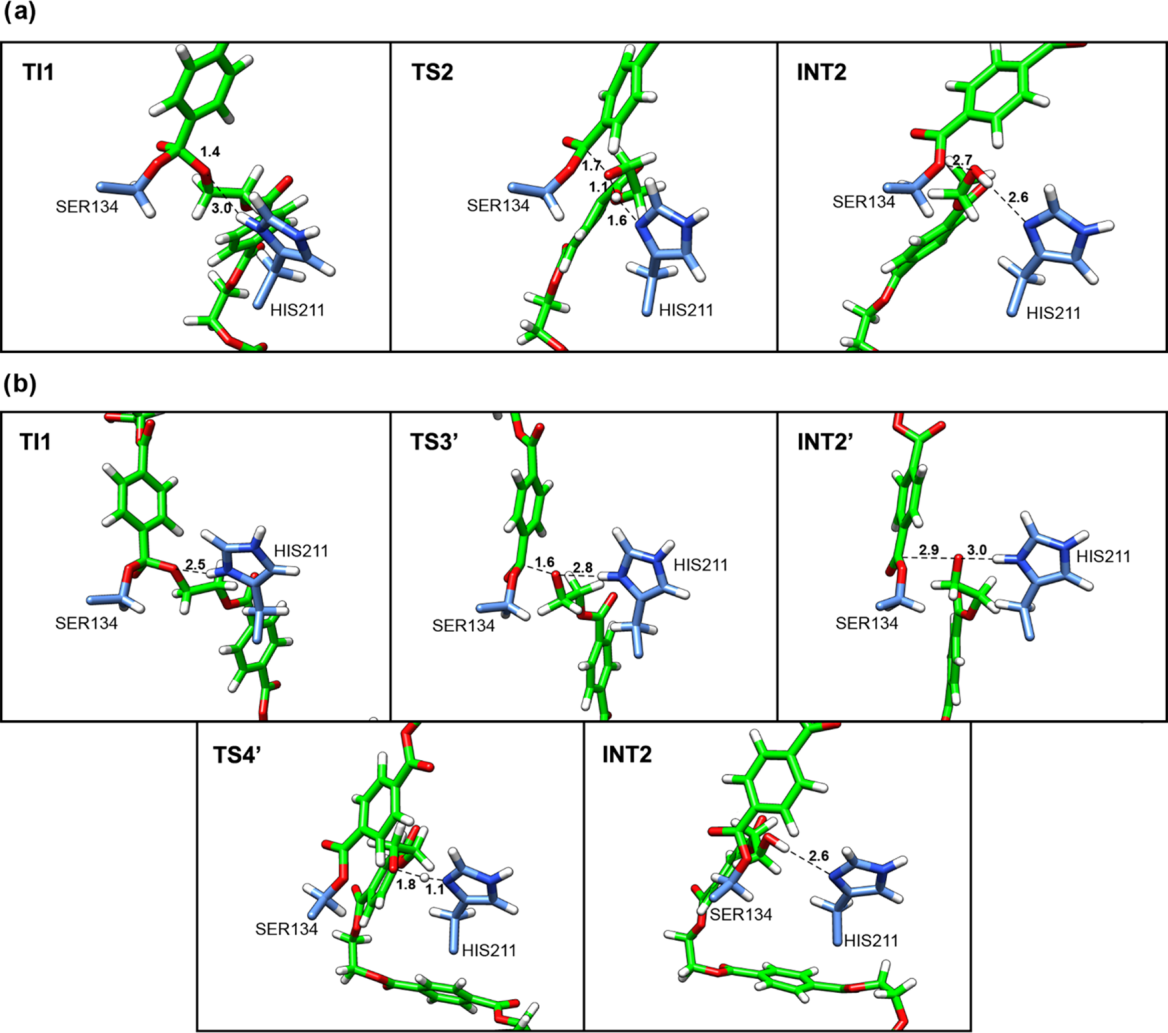
Representative structures along the 2D-PMF obtained from the QM/MM free-energy simulations for Step 2 acylation PET breakdown: (a) Path 1; (b) Path 2. Structures are labeled according to their locations along the 2D PMF.

Figure 4 shows the formation of an acyl-enzyme intermediate (INT2). There are also two possible paths on the FES to form this intermediate. Path 1 occurs through TS2. In the TS2, the R5 corresponds to 1.6 Å (see Figure 4a and Table S1). These coordinates represent the average distance between O_γ_ and the carbon atom (C_4_^1^), which is associated with the breaking of the ester bond of the PET (Figure 4a). The proton transfer from His211-NE1 to O_γ_ of PET is represented by the R2-R4 distances. In TS2, R4 corresponds to 1.1 Å (see Scheme 3, Table S1), indicating that proton transfer from His211-NE1 to O_γ_ of PET occurs first, then the cleavage of the ester bond of the PET takes place. This result shows another alternative mechanism to the previously described work for PET degradation by *Is*PETase,^20^ where first the proton transfer from His211 to PET occurs, and then PET cleavage takes place. The free energy of activation obtained at the AM1/d-PhoT/MM level corresponds to 13.8 kcal/mol for path 1 (Figure 2).

Path 2 is in accordance with the mechanism of PET degradation by *Is*PETase described in the previous contribution.^20^ In TS3′, the R5 corresponds to 1.8 Å and R2 is 1.0 Å (see Figure 4b and Table S2). These results show the advanced stage of the breaking of the ester bond of the PET in TS3′, while the proton remains connected to His211-NE1. The INT2 corresponds to the acyl-enzyme intermediate (Figures 1b and 4b). The path with lower barrier for reaction is through TS3′ with a free energy activation of 11.5 kcal/mol. In this step, there is the formation of an acetyl-enzyme intermediate (with Ser134 bonded to MHET), as also proposed in a previous computational study.^19^

**Figure 5:**
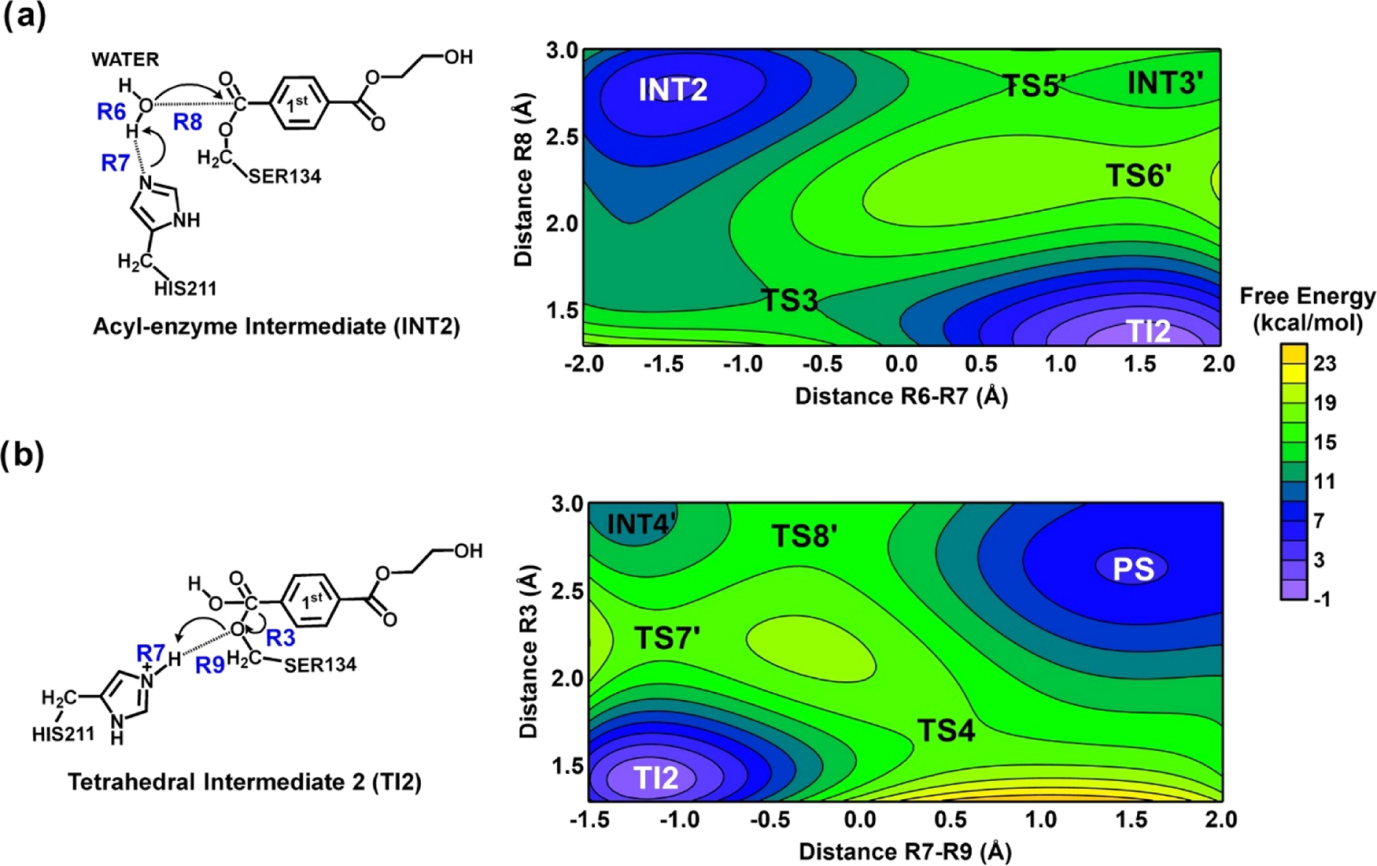
MHET Formation Mechanism PMF2D, describing the deacylation steps (a) Step 3; (b) Step 4 as described in Scheme 2. Contour interval of 2 kcal/mol.

**Figure 6:**
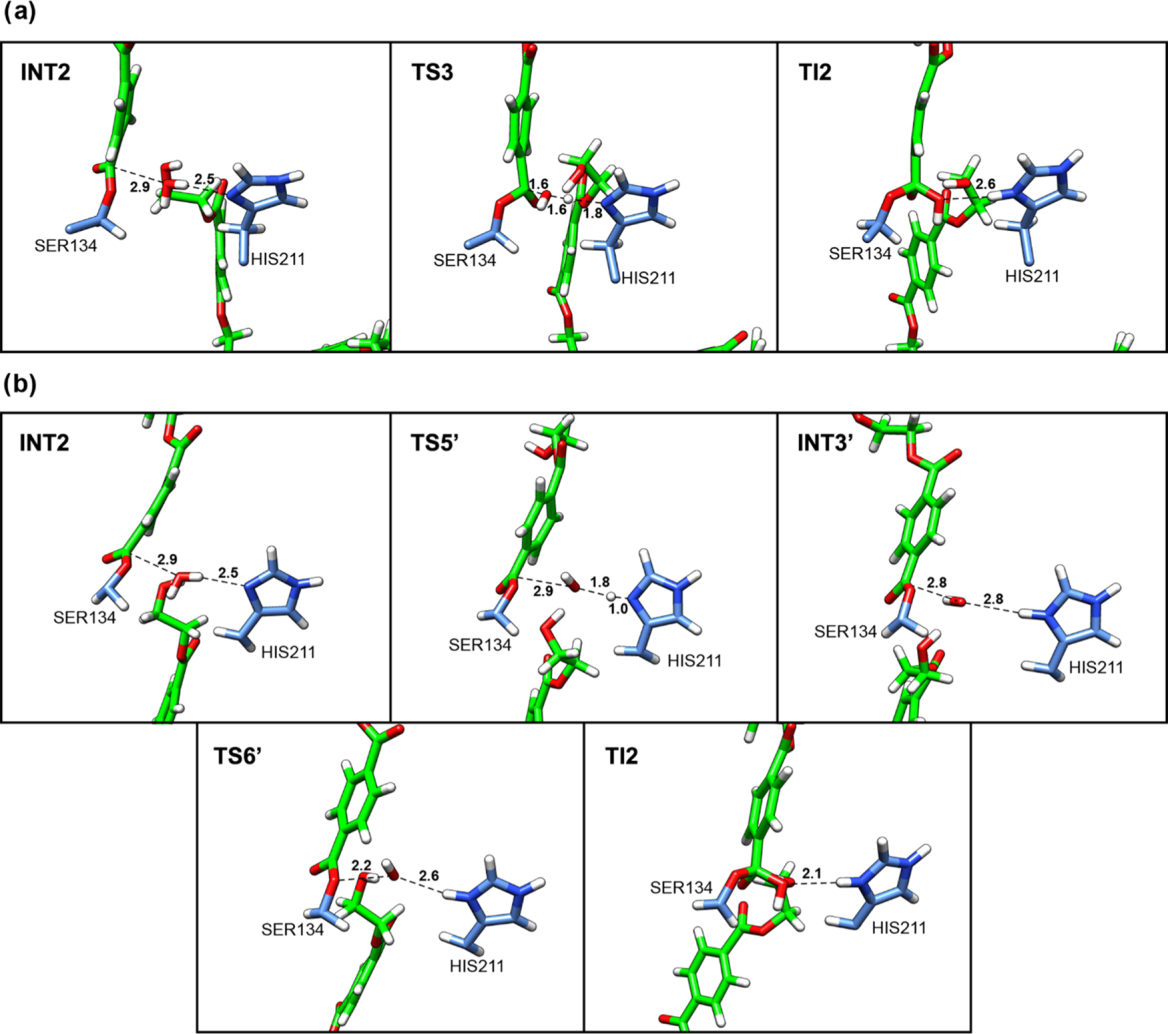
Representative structures along the 2D-PMF obtained from the QM/MM free-energy simulations for Step 3 deacylation water attack (a) concerted proposal; (b) stepwise proposal. Structures are labeled according to their locations along the 2D PMF.

Figure 5 shows the FES for deacylation, where a water molecule of the solvent plays a critical role. In step 3, the barrier calculated at AM1/d-PhoT/MM potential for path 1 is 8.3 kcal/mol. In TS3, the distance between the water oxygen and C_4_^1^ is 1.6 Å, while the R6 and R7 distances describing the proton transfer from water to His211 are 1.1 and 1.8 Å, respectively (Figure 6a and Table S3). The product on the free energy surface (Figure 5a) step corresponds to the tetrahedral intermediate 2 (TI2). The transition states for TI2 formation identified using AM1/d-PhoT/MM potential are similar to the ones found in previous studies.^45,46,47^ Path 2 occurs in a stepwise mechanism through TS5′ and TS6′ formation, with activation free energy of 10.3 and 3.6 kcal/mol, respectively (see Figure 2). In the TS5′, the average distance between the water molecule and carbon atom (C_4_^1^) corresponds to 2.9 Å, and the average distance between the proton of water and its oxygen is 1.8 Å, with the proton located closer to His211-NE1 (1.0 Å) (Figure 6b and Table S4). In TS6′, the average distance between the oxygen of the formed hydroxyl group and the carbon atom (C_4_^1^) corresponds to 2.2 Å. In the next step, the proton transfer from His211-NE1 to O_γ_ of MHET, represented by R7-R9, is associated with the breaking of the TI2 (R3 coordinate) (Figure 6b and Table S4).

**Figure 7:**
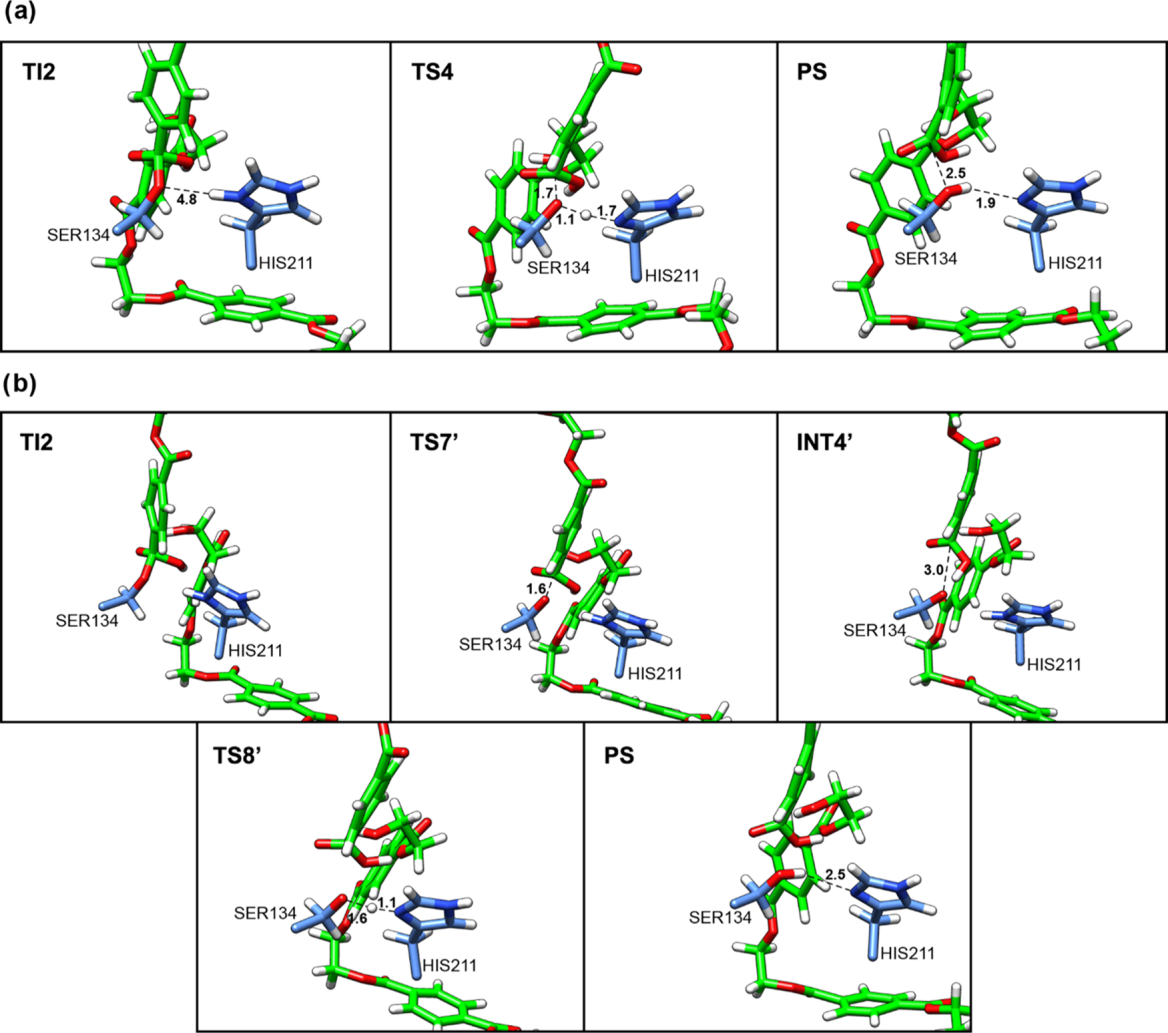
Representative structures along the 2D-PMF obtained from the QM/MM free-energy simulations for Step 4 deacylation tetrahedral intermediate breakdown: (a) concerted proposal; (b) stepwise proposal. Structures are labeled according to their locations along the 2D PMF.

On FES, the TI2 (tetrahedral intermediate 2) is at a clear minimum (see Figure 5). In TS4, the R3, R7, and R9 correspond to 1.7, 1.1, and 1.7 Å, which indicates the advanced stage of the proton transfer and the beginning of breaking the O_γ_-C_4_ bond of acylated Ser134 (Figure 7a). Path 1, as depicted in Figure 5a, outlines a concerted and asynchronous mechanism for this step, consistent with prior computational contributions.^20^ Path 2 leads to the formation of INT4′, where first occurs the O_γ_-C_4_ bond break of acylated Ser134, and then the proton transfer from His211 to INT4′ takes place (Figure 7b). Interestingly, the barriers of reaction for Path 1 and Path 2 are 16.1 and 16.4 kcal/mol, respectively. Therefore, based on these results, both paths are feasible.

The PS on this FES (Figure 5) corresponds to the reaction product, MHET. It is important to note that, according to our results, MHET is formed in the deacylation step. Initially, this may seem divergent from the result obtained in reference,^20^ where an MHET leaving group is observed in the acylation step. However, a thorough analysis of the system reveals that MHET formation may occur in both steps, depending on the polymer size used in simulations and the choice of ester carbon for nucleophilic attack. In our case, we used a PET tetramer, while reference^20^ used a PET dimer. This observation aligns with previous contributions, which highlight that the ester bond of the polymer is attacked by serine in the active site of PETase, forming a tetrahedral intermediate. Subsequently, the first product of the reaction is MHET or a fragment of the polymer, depending on the length of the initial polymeric chain and the site of cleavage. Then, an acyl-enzyme intermediate is generated.^19^ Therefore, the QM/MM with a reduced QM size and AM1/d-PhoT/MM potential describes the mechanism steps in agreement with experimental data.

The estimated experimental activation free energy for PET degradation (using transition state theory to obtain activation free energy from experimental turnover rate) is 18.0 kcal/mol.^7,9^ The uncertainty associated with this experimental value is less than 1 kcal/mol, considering the potential variability in the experimental conditions and measurement precision. We have determined that the reaction barriers for the last step of deacylation are 16.1 and 16.4 kcal/mol for paths 1 and 2, respectively (see Figures 2 and 5). For acylation, we have found barriers involved in the mechanism to be below 13.8 kcal/mol, in agreement with reference.^21^ In general, according to our results of the energy difference between the TS and its preceding intermediate (Figure 3), deacylation is the rate-limiting step of the process. Interestingly, Knott et al. have proposed that the deacylation step is the rate-limiting step in MHETase’s breakdown of an MHET molecule.^21^ Additionally, cutinase QM/MM simulations for PET biotransformation processes revealed that deacylation is the rate-limiting step of PET degradation.^50^ These results diverge from the observations reported in references^19^ and.^20^ On the other hand, considering the energy difference between each TS and RS (Figure 3), the acylation step can be regarded as the rate-limiting step in the PET degradation reaction by *Is*PETase. This step has a barrier of 13.2 kcal/mol (stepwise) or 13.8 kcal/mol (concerted), consistent with reference,^19^ which describes the reaction mechanism of PET hydrolysis by PETase and LCC enzymes using QM/MM MD simulations, finding a barrier of 14.6 kcal/mol for the deacylation step. Our results also align with Reference,^20^ which offers an atomistic and thermodynamic interpretation of the catalytic mechanism of PETase using umbrella sampling simulations at the PBE/MM MD level with a large QM region. This study details a two-stage reaction mechanism (acylation and deacylation), with the deacylation step having a free energy barrier of 15.1 kcal/mol.

### Energy Corrections

In general, the AM1/d-PhoT/MM potential described the species along the reaction profile, consistent with previous computational contributions. According to experimental data for PET degradation, the activation free energies are approximately 18 kcal/mol.^7,9^ Previous computational contributions found values of 4.7 kcal/mol,^51^ 17.8 kcal/mol,^46^ and 20.3^19^ kcal/mol for the activation free energy associated with the acylation step. Interestingly, reference^20^ reported values of 20 and 15 kcal/mol for acylation and deacylation, respectively. It is important to emphasize that we used a semiempirical Hamiltonian to model the quantum mechanics (QM) region. While this approach enables the simulation of free energy profiles in a timely manner, it is less accurate compared to *ab initio* and density functional theory (DFT) methods. Indeed, the semiempirical potential used to describe the QM region underestimates activation barriers compared to DFT for the acylation step. However, for deacylation, we observed remarkable agreement, with a deviation of only 1 kcal/mol from the reference.^20^

**Figure 8:**
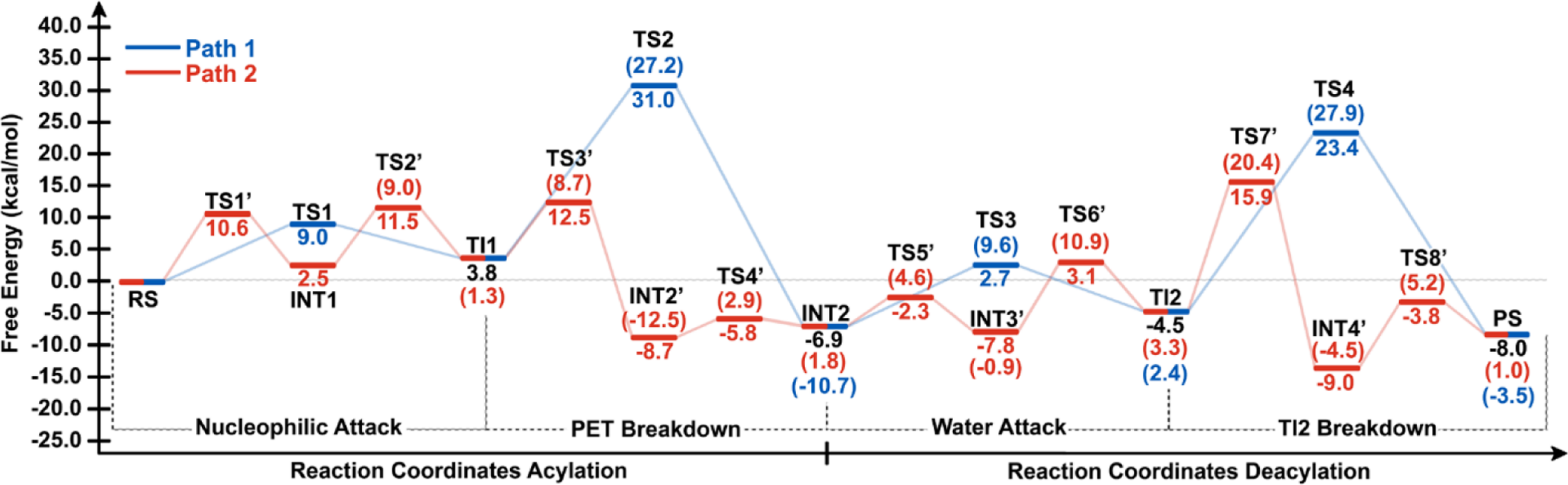
Free energies calculated at the M06-2*X*/6-31+G(d,p)/MM level for the MHET Formation. The values in parentheses represent the energy difference between the TS and its preceding intermediate, while the values outside parentheses are calculated with respect to the RS. Path 1 is in blue, and Path 2 is in red. Note that for some intermediates, two values are described for the stepwise (red) and the concerted (blue) paths. The units are in kcal/mol.

In order to correct the energies obtained with semiempirical potential, we applied corrections to the minimum free energy path by subtracting the energy calculated for the QM region using the semiempirical level and adding the energy from the M06-2*X*/6-31+G(d,p) level (Figure 8). The results reveal that acylation and deacylation take place in a stepwise manner, which is in remarkable agreement with previous computational contribution,^19^ where Path 2 for both acylation and deacylation presents the lower barrier for MHET formation (Figure 8). Energies calculated at M06-2*X*/6-31+G(d,p)/MM level showed that path 2 (Figure 8) describing the proton transfer from His211-NE1 to O_γ_ of PET has the lowest Δ*G*^‡^ value (2.9 kcal/mol), which suggests that it is the most energetically favorable step in the reaction under standard conditions. Also, the lowest Δ*G* value is seen in path 2 during PET breakdown (−12.5 kcal/mol), indicating that it is the most energetically favorable step under actual reaction conditions. Step 3 has the highest Δ*G* value (3.3 kcal/mol), suggesting that it is the least energetically favorable step under actual reaction conditions. Step 4 has the highest Δ*G*^‡^ value (27.9 kcal/mol for the concerted path and 20.4 kcal/mol in the first step of the stepwise path), which indicates that it is the least energetically favorable step under standard conditions. Overall, the rate-limiting step was found to be deacylation with a barrier of 20.4 kcal/mol, which is in agreement with the computational value of 19.8 kcal/mol.^21^ It is worth noting that, considering the energy difference between the TS and RS calculated at the M06-2*X*/6-31+G(d,p)/MM level for MHET formation, the deacylation step is also the rate-limiting step in the reaction involving PET and *Is*PETase (see Figure 8).

### Per-residue Interaction Energy

Here, we employed residual decomposition analysis to explore the contributions of individual molecular components to the overall electrostatic interaction energy between PET tetramer and *Is*PETase at the reactants (RS) and transition states (TS) (Figure 8). This approach yields insights into the specific residues that can either stabilize or destabilize the TS.

**Figure 9:**
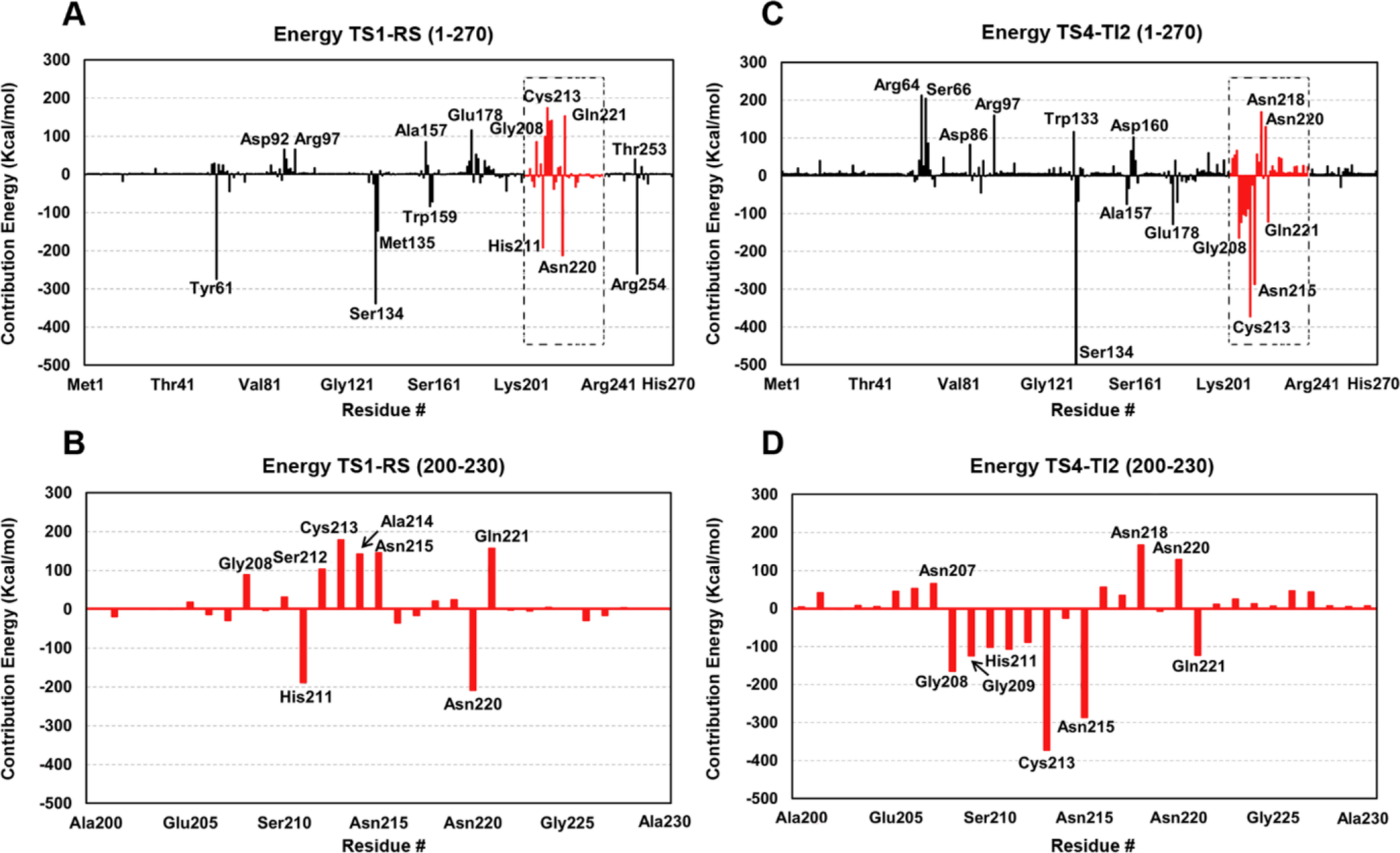
Per-residue interaction energy contributions of individual amino acids to complex stabilization of TS1 and TS4 from the PETase mechanism calculated at the M06-2*X*/6-31+G(d,p)/MM level (in kcal/mol) (a) TS1-RS; (b) TS1-RS highlighting the residues 200–230 (c) TS4-INT3; and (d) TS4-INT3 highlighting the residues 200–230. Interaction energies larger than 60 kcal/mol are labeled.

Positive values indicate energy contributions with repulsive interactions with the transition state, whereas negative values suggest contributions favoring stabilization. Upon analysis of these results, in the first step of acylation, several residues display notable effects on the TS1. The residues Tyr61, Asp92, Arg97, Ser134, Met135, Ala157, Trp159, Glu178, Gly208, His211, Cys213, Gln221, Asn220, Thr253, and Arg254 show significant energy contributions, either stabilizing or destabilizing the transition state (Figure 9a). These residues play a crucial role in catalysis or structural stabilization during the enzymatic reaction between PET and *Is*PETase.

We highlight the residues located between 200 and 230 positions in *Is*PETase with large positive energies, such as Gly208, Ser212, Cys213, Ala214, Asn215, and Gln221 (Figure 9c). These residues might represent key positions for structural modifications or targeted mutations to reduce the reaction barrier during this step and enhance the enzyme’s efficiency or selectivity in the catalytic process. For the last step of the reaction, in the TI2 breakdown and therefore MHET release, the residues Arg64, Ser66, Arg97, Trp133, Ser134, Asp160, Glu178, Gly208, Cys213, Asn215, Asn218, Asn220, and Gln221 show significant energy contributions, either stabilizing or destabilizing the transition state (Figure 9c). The high energy contribution of Ser134 is related to its covalent bond with the substrate. Again, a cluster of residues with high interaction can be seen in the region 200 to 230 (Figure 9d), but differently from Step 1, we have seen a great number of attractive interactions with residues Gly208, Gly209, His211, Cys213, Asn215, and Gln221 playing an important role in the MHET release.

**Figure 10:**
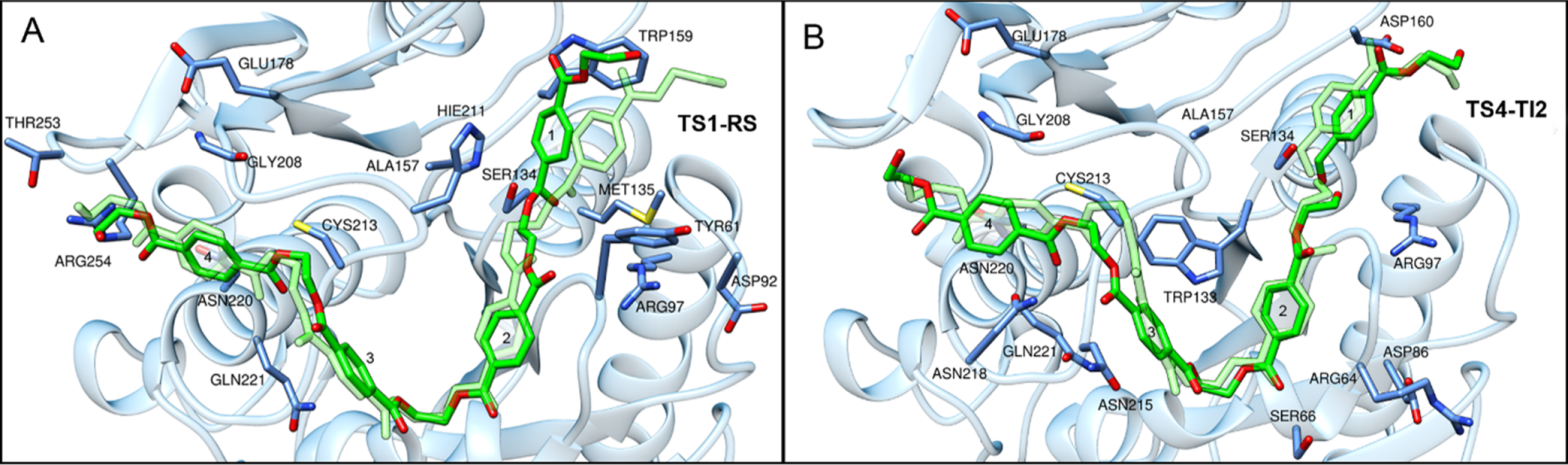
Transition State Analysis of *Is*PETase Enzyme Catalysis. (a) TS1 Acylation Step with Key Residues from Per-Residue Interaction Energy Analysis Highlighted; (b) TS4 Deacylation Step with Corresponding Residue Interactions. The PET tetramer is green, while the key residues and protein are blue. The transparent green color represents the RS and TI2 PET conformations.

Residues such as Arg254, Tyr61, and Cys213 exemplify the nuanced interplay of repulsive and attractive contributions in transition state stabilization. Arg254′s notable decrease in energy contribution (−260.11 kcal/mol) could reflect a reduction in electrostatic repulsion during TS1 formation, which is critical for stabilizing the PET subunit 4 that is located far away from the active site. This indicates Arg254′s pivotal role in neutralizing repulsive forces that could help stabilize the transition state. Similarly, a drastic energy decrease is observed for Tyr61 (−274.20 kcal/mol), which could be a result of the coordination of the PET monomer 1, due to the significant movements required in the first steps of the reaction (Figure 10a). Cys213′s significant energy increase (176.47 kcal/mol) suggests a shift toward repulsive interactions, likely due to its role in orienting the substrate as it is strategically positioned between subunits 3 and 4, thus highlighting its essential contribution to maintaining a favorable energetic environment for the transition state. On the other hand, during the formation of TS4, its role is inverted as the movement of the end chain of PET is considerably larger than in the first steps of the mechanism (Figure 10b). Figure 10 depicts the transition states’ most significant residues, obtained by decomposition analysis during the PETase acylation and deacylation steps. These steps are critical because they involve the formation of a covalent acyl-enzyme intermediate and the release of the product, a key component of the enzyme’s catalytic mechanism.

In addition to the interactions that define the central role of the catalytic serine and histidine residues, these findings highlight the presence of aromatic residues, such as tryptophan and tyrosine, which stabilize both transition states and provide a hydrophobic environment suitable for catalysis. However, their exact contributions may differ, with Trp159 in TS1 and Trp133 in TS4, indicating subunit-specific interactions with the substrate. The differences in the electrostatic environment between TS1 and TS4 provide insight into the fine-tuning required for each step. TS1, for example, contains Arg97 and Met135, whose contributions may not be absent in TS4, implying a distinct electrostatic and structural environment for the acylation step. In contrast, TS4 has residues such as Asn220 and Gln221, indicating a unique network of hydrogen bonds important for the deacylation process.

Electrostatic contributions to PETase TS1 are mostly concentrated in the first subunit, but in TS4, these contributions move to the third and fourth subunits. These differences in the electrostatic interactions during the reaction could be attributed to the preparation of the PETase for nucleophilic attack in the first step and the product release in the final step of the reaction.

The residual decomposition analysis further refines our understanding of the catalytic mechanism by isolating the contributions of individual amino acid residues to the total electrostatic interaction energy. This approach not only aids in identifying key residues that stabilize or destabilize transition states and intermediates, but also provides new opportunities for enzyme engineering by pinpointing targets for mutation or structural modification. These findings are helpful for creating enzymes with improved catalytic capabilities, as seen by the important roles that residues like Tyr61, Ser134, and His211 play in stabilizing transition states. Recent studies have emphasized the crucial role of noncovalent interactions in maintaining the three-dimensional structure and function of proteins.^52^ With our decomposition analysis, we could observe that this flexibility is particularly evident in the acylation step of PETase, where interactions between Met135, Tyr61, and the first ring of the PET polymer were crucial for the stabilization of the oxyanion (Figures 8a and 10a). The interaction contribution of each residue can be found in Supporting Information.

Simulation and experimental data are useful for machine learning methods applied in the discovery and annotation of promising enzymes, as well as in suggesting beneficial mutations for improving known targets.^53,54^ Indeed, combining experimental data on mutations and catalysis^55−57^ with the computational interaction energies of different residues and their contributions to catalysis using machine learning (ML) algorithms may offer opportunities to design new, more effective catalytic systems. By analyzing the electrostatic profiles during catalysis obtained from QM/MM of enzymes such as PETase, we aim to create a ML model to predict the electrostatic impacts of mutations to predict beneficial mutations, optimize enzyme design for improved catalytic properties, and accelerate the discovery of efficient enzyme variants. Additionally, it is possible to develop a comprehensive mutation database from MD simulations and QM/MM calculations. This integrated approach may accelerate the pace of enzyme engineering, opening new possibilities for creating catalytic systems specifically designed to degrade synthetic polymers like PET.

## Conclusions

We used the QM/MM approach to investigate PET degradation by *Is*PETase, providing a detailed analysis of the energetics involved in the formation of the product mono(2-hydroxyethyl) terephthalic acid (MHET). The acylation step simulations, focusing on the formation of tetrahedral intermediate 1 (TI1), unveiled two potential paths: a concerted mechanism through TS1 and a stepwise pathway via TS1′ and TS2′. The FES results indicated an endergonic reaction for the formation of TI1, aligning with prior computational insights. Further exploration into the deacylation process elucidated the formation of tetrahedral intermediate 2 (TI2), a critical stage where a water molecule intervenes in the PET breakdown. Furthermore, residual decomposition analysis identified specific amino acid residues that contribute to stabilization or destabilization of the transition states. Our investigation highlighted that a cluster of residues, particularly in the 200–230 region, exhibited markedly positive energy contributions during the acylation, implying a potential site for structural modifications or targeted mutations. On the other hand, the same region provided negative energy contributions during the last step of the reaction. Overall, the energetic values obtained from our 2D-PMF for the acylation step using QM/MM potential are consistent with previous studies. This result can be used for exploring the mechanism of PET hydrolysis with less computational cost compared with previous simulations, which could be an important tool for designing *Is*PETase variants if one must explore key mutations that would affect the activation energy. Understanding the degradation of PET into its monomeric components is crucial for the engineering of enzyme systems tailored for PET degradation.

## Supporting Information Available

Oxyanion hole formed by Tyr61 and Met135 during acylation; transition state structure for TS2; TS1′, TS3′, TS4′; average key distances (Å) for structures obtained from 2D-PMF; methods, softwares, and PDB structures (PDF)
Per-residue interaction energy calculated at the M06-2*X*/6-31+G(d,p)/MM level (XLS)
