# Supplementary material for: Exploring the Reaction Mechanism of Polyethylene Terephthalate Biodegradation through QM/MM Approach
Source: J Phys Chem B. 2024 Jul 29;128(31):7486–99. doi: 10.1021/acs.jpcb.4c02207 (PMC11317977; doi:10.1021/acs.jpcb.4c02207)
Supplement: Supplementary file 1 — jp4c02207_si_001.pdf [file jp4c02207_si_001.pdf]

Supporting Information

Exploring the Reaction Mechanism of Polyethylene terephthalate Biodegradation through QM/MM Approach

Alberto M. dos Santos,[1] Clauber H. S. da Costa,[1] Pedro H. A. Silva,[2] Munir Skaf,[1] Jerônimo Lameira[2*]

[1]*Institute of Chemistry and Center for Computer in Engineering and Sciences, University of Campinas (UNICAMP), Campinas 13084-862, Sao Paulo, Brazil*

[2]*Institute of Biological Sciences. Federal University of Pará, 66075-110, Belem, Para, Brazil.*

*correspondence should be addressed:

J. Lameira: lameira@ufpa.br

Representative PDB structures

In addition, we have a publicly available the representative pdb structures on GitHub (https://github.com/albertomds/petaseMechanism).

Table S1. Average Key Distances (Å) for Structures Obtained from 2D-PMF for Acylation Step concerted proposal. Reaction coordinates R1–R11 defined in Scheme 2 and Standard Deviations Given in Parentheses.

distances	RS	TS1	TI1	TS2	INT2
**R1**	1.01 (0.04)	1.09 (0.03)	2.65 (0.08)	2.47 (0.12)	3.14 (0.22)
**R2**	2.59 (0.09)	1.82 (0.07)	1.03 (0.03)	1.64 (0.06)	2.60 (0.07)
**R3**	2.90 (0.08)	1.58 (0.04)	1.44 (0.03)	1.39 (0.02)	1.36 (0.02)
**R4**	3.20 (0.31)	2.34 (0.14)	2.97 (0.30)	1.11 (0.04)	0.99 (0.02)
**R5**	1.39 (0.02)	1.43 (0.03)	1.47 (0.03)	1.65 (0.06)	2.70 (0.07)
**R6**	0.99 (0.03)	1.00 (0.03)	1.00 (0.03)	0.98 (0.04)	0.98 (0.03)
**R7**	4.95 (0.26)	5.56 (0.18)	6.20 (0.21)	7.61 (0.43)	9.39 (1.13)
**R8**	3.04 (0.16)	3.03 (0.12)	3.16 (0.11)	5.51 (0.37)	5.14 (0.71)
**R9**	2.11 (0.16)	3.01 (0.18)	3.31 (0.20)	4.98 (0.47)	5.54 (1.07)

Table S2. Average Key Distances (Å) for Structures Obtained from 2D-PMF for Acylation Step stepwise proposal. Reaction coordinates R1–R11 defined in Scheme 2 and Standard Deviations Given in Parentheses.

distances	RS	TS1’	INT1’	TS2’	TI1	TS3’	INT2’	TS4’	INT2
**R1**	1.01 (0.04)	1.77 (0.07)	3.16 (0.08)	2.91 (0.09)	2.65 (0.08)	4.17 (0.20)	4.65 (0.15)	4.39 (0.20)	3.14 (0.22)
**R2**	2.59 (0.09)	1.07 (0.02)	1.09 (0.03)	1.05 (0.04)	1.03 (0.03)	1.04 (0.02)	1.03 (0.02)	1.08 (0.04)	2.60 (0.07)
**R3**	2.90 (0.08)	2.94 (0.07)	3.06 (0.08)	2.29 (0.09)	1.44 (0.03)	1.41 (0.02)	1.37 (0.02)	1.36 (0.02)	1.36 (0.02)
**R4**	3.20 (0.31)	3.44 (0.27)	4.41 (0.22)	3.44 (0.31)	2.97 (0.30)	2.79 (0.08)	2.96 (0.09)	1.77 (0.07)	0.99 (0.02)
**R5**	1.39 (0.02)	1.39 (0.02)	1.39 (0.02)	1.39 (0.03)	1.47 (0.03)	1.64 (0.06)	2.92 (0.08)	2.89 (0.07)	2.70 (0.07)
**R6**	0.99 (0.03)	0.99 (0.03)	0.99 (0.03)	0.99 (0.02)	1.00 (0.03)	0.99 (0.03)	0.99 (0.04)	0.99 (0.04)	0.98 (0.03)
**R7**	4.95 (0.26)	4.29 (0.22)	5.81 (0.29)	5.76 (0.23)	6.20 (0.21)	13.54 (1.03)	13.96 (0.84)	12.61 (1.55)	9.39 (1.13)
**R8**	3.04 (0.16)	2.99 (0.15)	3.03 (0.16)	2.94 (0.12)	3.16 (0.11)	10.17 (0.96)	9.70 (0.57)	8.55 (1.58)	5.14 (0.71)
**R9**	2.11 (0.16)	1.97 (0.15)	2.12 (0.26)	2.47 (0.24)	3.31 (0.20)	10.29 (1.16)	9.47 (0.80)	9.48 (1.74)	5.54 (1.07)

Table S3. Average Key Distances (Å) for Structures Obtained from 2D-PMF for Deacylation Step concerted proposal. Reaction coordinates R1–R11 defined in Scheme 2 and Standard Deviations Given in Parentheses.

distances	INT2	TS3	TI2	TS4	PS
**R1**	3.22 (0.28)	3.88 (0.12)	3.77 (0.10)	4.47 (0.23)	4.71 (0.30)
**R2**	4.70 (0.32)	3.63 (0.34)	3.90 (0.40)	5.57 (0.29)	6.11 (0.40)
**R3**	1.36 (0.02)	1.41 (0.02)	1.42 (0.02)	1.73 (0.11)	1.89 (0.11)
**R4**	0.99 (0.02)	0.99 (0.02)	0.99 (0.03)	0.98 (0.02)	0.98 (0.02)
**R5**	3.05 (0.07)	3.14 (0.08)	3.09 (0.07)	3.08 (0.08)	3.07 (0.07)
**R6**	0.99 (0.03)	1.56 (0.04)	2.55 (0.09)	2.32 (0.17)	2.42 (0.18)
**R7**	2.51 (0.08)	1.79 (0.07)	1.03 (0.04)	1.68 (0.07)	2.53 (0.08)
**R8**	2.88 (0.07)	1.57 (0.05)	1.45 (0.03)	1.38 (0.02)	1.37 (0.02)
**R9**	3.50 (0.15)	3.21 (0.09)	4.78 (0.11)	1.10 (0.04)	1.01 (0.02)

Table S4. Average Key Distances (Å) for Structures Obtained from 2D-PMF for Deacylation Step stepwise proposal. Reaction coordinates R1–R11 defined in Scheme 2 and Standard Deviations Given in Parentheses.

distances	INT2	TS5’	INT3’	TS6’	TI2	TS7’	INT4’	TS8’	PS
**R1**	3.22 (0.28)	2.82 (0.20)	3.40 (0.16)	3.60 (0.16)	3.77 (0.10)	4.40 (0.26)	3.46 (0.38)	4.46 (0.51)	4.71 (0.30)
**R2**	4.70 (0.32)	4.09 (0.21)	4.91 (0.21)	4.84 (0.37)	3.90 (0.40)	5.49 (0.29)	4.70 (0.49)	5.11 (0.77)	6.11 (0.40)
**R3**	1.36 (0.02)	1.36 (0.02)	1.37 (0.03)	1.38 (0.04)	1.42 (0.02)	1.62 (0.05)	2.98 (0.07)	2.93 (0.07)	1.89 (0.11)
**R4**	0.99 (0.02)	1.02 (0.05)	1.00 (0.03)	1.00 (0.02)	0.99 (0.03)	0.98 (0.02)	0.99 (0.02)	0.99 (0.05)	0.98 (0.02)
**R5**	3.05 (0.07)	3.04 (0.08)	3.09 (0.07)	3.11 (0.08)	3.09 (0.07)	3.09 (0.07)	3.11 (0.08)	3.04 (0.08)	3.07 (0.07)
**R6**	0.99 (0.03)	1.84 (0.06)	2.76 (0.10)	2.56 (0.08)	2.55 (0.09)	2.13 (0.16)	2.80 (0.28)	2.75 (0.15)	2.42 (0.18)
**R7**	2.51 (0.08)	1.04 (0.02)	1.03 (0.03)	1.03 (0.02)	1.03 (0.04)	1.05 (0.02)	1.02 (0.02)	1.11 (0.03)	2.53 (0.08)
**R8**	2.88 (0.07)	2.94 (0.07)	2.84 (0.08)	2.19 (0.28)	1.45 (0.03)	1.40 (0.02)	1.31 (0.02)	1.32 (0.02)	1.37 (0.02)
**R9**	3.50 (0.15)	4.50 (0.17)	4.68 (0.19)	4.45 (0.21)	4.78 (0.11)	2.21 (0.08)	2.34 (0.08)	1.63 (0.06)	1.01 (0.02)

Table S5. Free Energies calculated at M06-2X/6-31+G(d,p)/MM level for MHET Formation (kcal/mol).

Step	Path 1		Path 2			
**Acylation**	**ΔG‡**	**ΔG**	**ΔG1‡**	**ΔG1**	**ΔG2‡**	**ΔG2**
**Step 1 – Nucleophilic Attack**	9.0	3.8	10.6	2.5	9.0	-2.5
**Step 2 – PET Breakdown**	27.2	-0.4	8.7	-12.5	2.9	-4.7
**Deacylation**	**ΔG‡**	**ΔG**	**ΔG1‡**	**ΔG1**	**ΔG2‡**	**ΔG2**
**Step 3 –Water Attack**	9.6	-4.4	4.6	-0.9	10.9	-3.5
**Step 4 – TI2 Breakdown**	27.9	4.4	20.4	-4.5	5.2	8.8

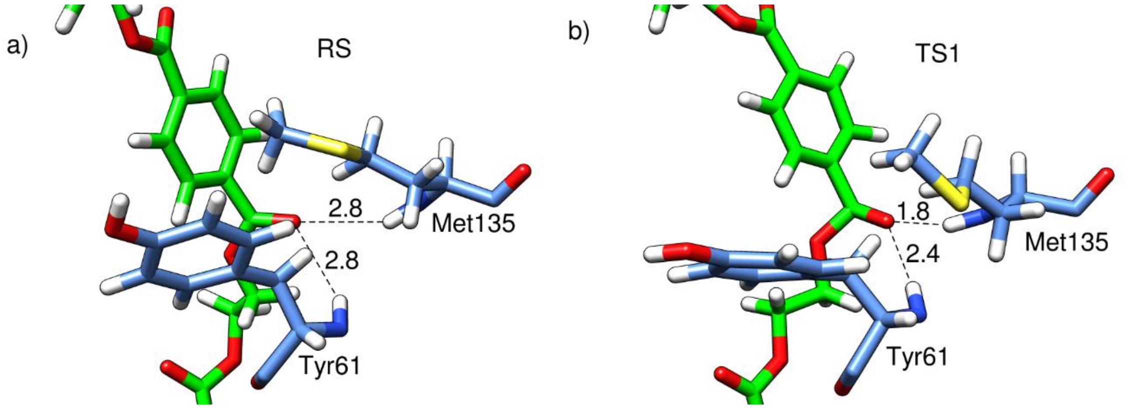

Figure S1. Oxyanion hole formed by Tyr61 and Met135 during acylation. a) RS structure b) TS1 structure obtained from path1.

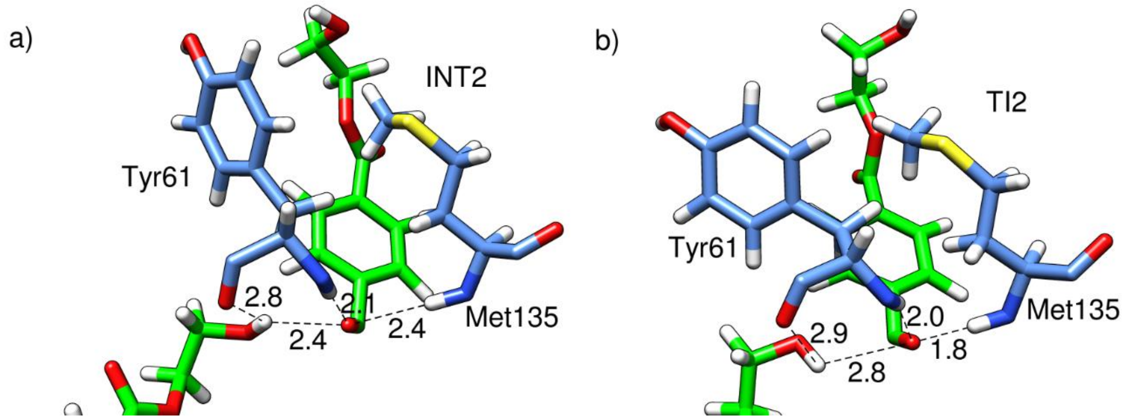

Figure S2. Oxyanion hole formed by Tyr61 and Met135 during acylation a) INT2 structure b) TI2 structure obtained from path1.

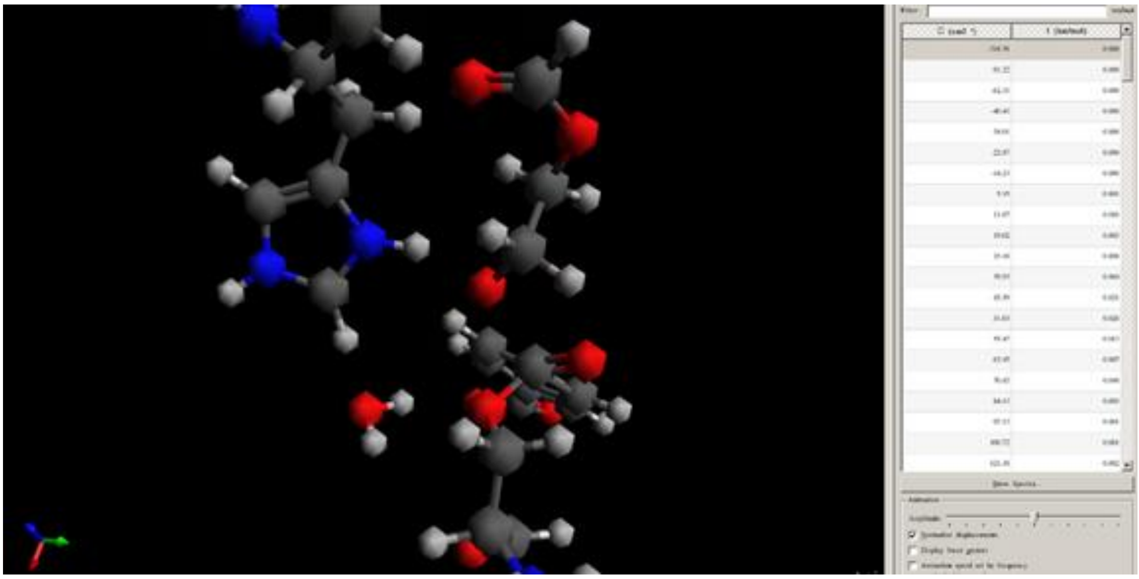

Figure S3. Transition state structure for TS2 and its calculated frequencies. Refer to Table S1 and the text of the main article for information about this step.

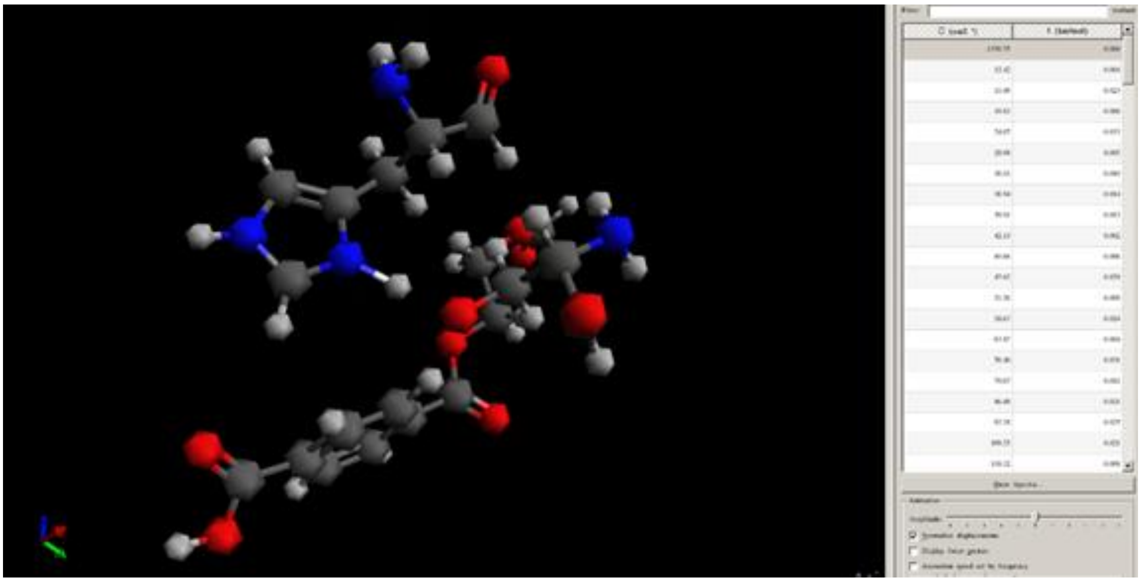

Figure S4. Transition state structure for TS1’ and its calculated frequencies. Refer to Table S2 and the text of the main article for information about this step.

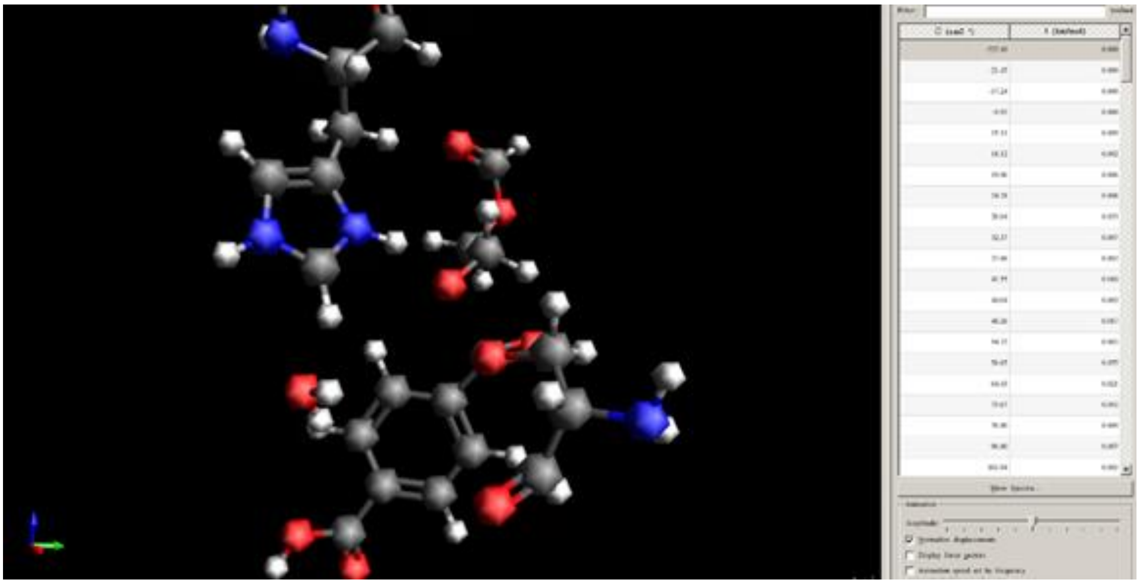

Figure S5. Transition state structure for TS3' and its calculated frequencies. Refer to Table S2 and the text of the main article for information about this step.

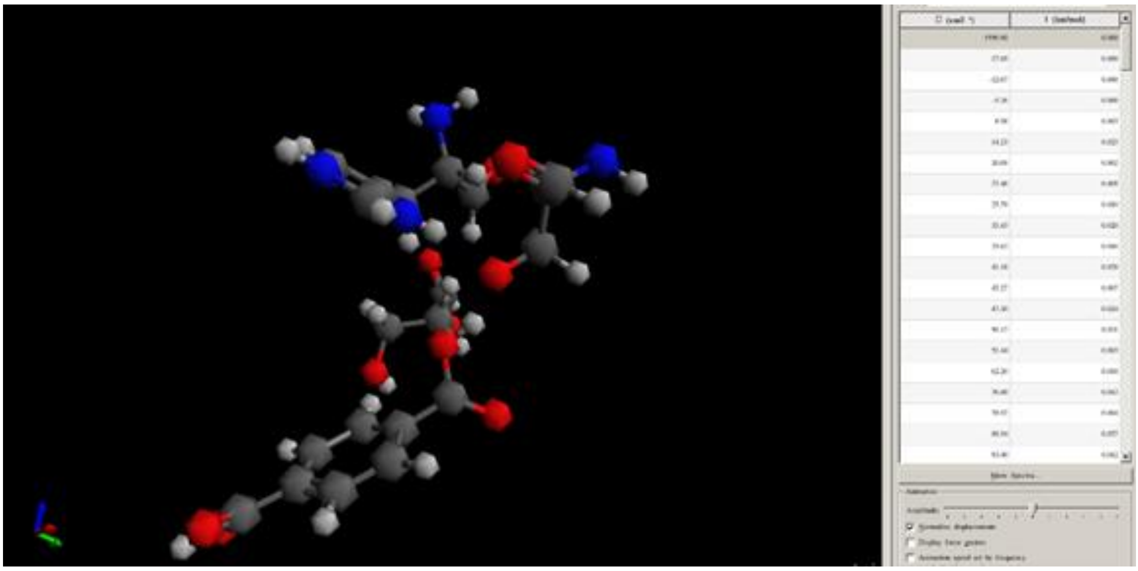

Figure S6. Transition state structure for TS4' and its calculated frequencies. Refer to Table S2 and the text of the main article for information about this step.
